# Southern Cattle Plague

**Published:** 1892-10

**Authors:** Frank S. Billings

**Affiliations:** Director of the Patho-Biological Laboratory of the State University of Nebraska


### THE ETIOLOGY OF SOUTHERN CATTLE PLAGUE—TEXAS FEVER.

(*Continued*).

By Frank S. Billings.

*Director of the Patho-Biological Laboratory of the State University of Nebraska.*

### THE BACILLUS OF SOUTHERN CATTLE PLAGUE IN THE TICKS.

Nearly every falsehood or misstatement has to have a microscopic fragment of truth in close proximity if it is to have any vitality whatever.

That is true of the germ claimed by the Government to be the specific cause of a second widespread plague in our hogs. The germ is ubiquitous. Of itself it seems to be harmless. As it has not been found alone and by itself causing the death of a single animal by the Government since it first announced the second plague in 1886. The true germ was in, or had been in the same hog, and prepared a feeding ground for the fraudulent germ. That is all they have proven. The same is true of the ticks. In fact several factors are often present together. Southern cattle, Southern ticks, and the disease in northern cattle. But the ticks are not present in all cases on our northern cattle. There were no ticks present on the cattle in the second outbreak at Tekamah, Neb., in 1887, and none on the grey steer at Roca, though on the other one. The dictum, "No Ticks no Texas Fever," will not work. It should read "no Sporozoa taken on Tick." Having demonstrated, beyond the power of man to successfully or honestly contradict, the presence of a specific bacillus in the blood and organic tissues of cattle afflicted with the southern cattle plague, it now becomes my most agreeable duty to show to the world the real part which the ticks can play in the historic genesis of this American cattle pest. While we shall speak of ticks, no one need take our "notes on tick." Their endorsement, with facts, will be so strong that they will be taken "at par" by any intelligent microbe broker in the world.

There is nothing new in the fact that southern cattle are frequently, if not generally, infested with ticks when they come North in the summer months. There is nothing new in the hypothesis that the "ticks" might possibly be in some way connected with the etiology of this disease. The assumption is comparable to that of corn-smut being the cause of the corn-stalk disease. The smut in that, the tick in this disease, were something tangible, and that which is before the eye and most marked is generally the thing blamed and the real sinner is seldom seen. The tick hypothesis really has more solid foundation to stand on before the Government united with it its absolutely ridiculous protozoön theory than it has now. But as in the "smut" assumption there was, and is something in corn causing disease, so in the tick hypothesis there is a truth which the people in Washington have either not seen or wilfully hidden from public view. The ease with which the thing was developed in our hands would seem to justify either malicious neglect or dishonesty on the part of the Government investigators.

### THE HISTORY OF ONE TICK AND WHAT IT REVEALED.

The fate of a battle has often been decided by a single shot. The fate justly due the investigators in Washington may result from the investigation of a single tick.

On Sunday, Oct. 4, 1892, I received a box of ticks from Dr. Tait Butler, veterinarian to the agricultural experiment station of Missisippi, the ticks having been picked off the cattle of the station, put in a box and sent me. The box was an oblong wooden one, with a wooden cover and not over clean inside. In it were quite a number of the large, greyish ovoid bugs known as Southern Cattle Ticks. The weather was unusually and desperately cold for the time of year. They were at once put in the Thermostat to see if it would not revive them, but it did not seem to. They were all shriveled up the next morning except one, which was still a full, plump and rather lively individual for a tick under such abnormal conditions.

In the box were, besides the ticks, roundish, irregularly surfaced masses, somewhat like grape-shot, about the size of a small pea, of a brick-red color, and looked like masses of stuff made in pellets and then stuck together. The stuff was the fæcal discharges of the ticks. There were no ova about them. It was not the season of the year for them perhaps. I might as well say that I did not believe much in the tick business any more than I did in the "smut" hypothesis in the corn-stalk disease, and had it not been for numerous friends around me at the time, I think it very probable, ticks, box, fæcal discharge and all would have gone into the fire. In order to gratify my friends' curiosity, I first consented to examine the "little red balls," as one called them, so one was broken open and a small piece taken from the centre, and rubbed upon a cover-glass with sterilzed water, and then stained with fuchsin and put under the microscope. My indifference was at once changed to most intense interest, for in this stuff there were millions of an ovcid belted germ so exactly like those I had obtained from every outbreak of Texas Fever that no mortal would ever have doubted their identity, knowing that these ticks also come from southern cattle. It will be remembered that southern cattle are seldom, if ever, ill themselves when capable of disseminating a death-dealing influence to our northern stock.

The next step was to examine the ticks, the plump, lively one being selected. The tick was first given a plunge-bath in corrosive-sublimate solution, then one in alcohol and a final one in sterilized water; it then had its back split open with sterilized scissors and out of the wound oozed a reddish-black thick stuff of tar-like consistency. Smears of this material were at once made and an apparently pure condition of the same germ that prevailed in the fæces observed. Cultures in bouillon gelatine, on agar and plates were made from this stuff, and most singular to say, were so pure that no other germ could be detected except a small rod and a coccus in three or four colonies each on the four plates. Plates were then made from the bouillon-gelatine and agar and with approximately the same result. These bouillon flasks and several gelatine tubes were pure, the agar had a few other colonies as above mentioned. The red-balled fæcal discharge, from six of the masses, a small fragment taken from the center being used, were then made in the same media, with approximately the same result. All other germs but the one mentioned were discarded. The germs thus isolated corresponds in every particular with the one obtained in previous investigations from the blood and organs of cattle peremptorily killed in order to make studies in the southern cattle plague. There is no need of repeating the descriptions.

Here then we have several positive discoveries as against the negative assertion of the Government that "Texas fever is a disease not caused by the bacteria," etc.

1st. Bacteria found in every outbreak of the disease in northern cattle to which we have had access or from which material has been sent us.

2d. The disease produced in cattle by inoculation with the germ as obtained from cattle.

3d. An identical germ found in the abdominal contents and and fæcal discharge of ticks direct from southern cattle.

The results conclusively demonstrate either insufficient investigation on the part of the Government investigators, or the suppression of the true facts.

But we all know that germs from two different sources can look alike and grow so very nearly alike as to be quite deceptive—though it must not be forgotten for an instant, that in these cases *we have a common origin for the germs.*

1st. In the blood and organs of cattle infected on pastures previously infested by Texas cattle, and in a steer inoculated with pure cultures from that steer.

2d. In ticks and other discharges which had been taken directly from the bodies of southern cattle.

The point to be at once decided, and in such cases as this I do not allow much dust to collect, so as to at once see what would be the effect of

### THE INOCULATION OF NORTHERN CATTLE WITH PURE CULTURES FROM THE TICK.

August 8, 1891, 11 A.M. I inoculated two calves, one a six months old Jersey, tough, rugged and stunted; the other smaller, a six weeks old good shorthorn, fat and sleek. Both received 10 ccms. of a pure bouillon culture of the same organism derived from the tick into the jugular vein.

4 P.M. Also inoculated a full grown guinea pig ½ ccm. and a full grown rabbit 1. ccm.—same bouillon culture. Made plates from bouillon used in these inoculations, the result gave pure cultures; carried all the various cultures in and on different media for some time, and then reduced them to bouillons, using gelatine cultures and occasional plates with a test of virulence, in guinea pigs occasionally.

9th. Guinea pig died at 5 P.M., blood full of the ovoid belted germ, pure cultures from heart's-blood.

11th. Rabbit died this morning. The big dose 1. ccm. in the rabbit, and the quick death of the guinea pig and slow course in the rabbit, gives another confirmatory point between this organism and the one found in cattle. Rabbits are not immune against the action of this germ, though so to the same doses used in ground squirrels and guinea pigs, but all my inoculations show this can be overcome by unreasonably large doses. The examination of the heart's blood of our rabbit and plate and other media of cultivation demonstrated no other germ present in the blood than the one injected.

THE RESULT OF THE TICK-GERM INOCULATION IN THE CALVES.

September 9th, 3 P.M. Went out to the farm at the request of the Superintendent, who reported the germ injected to be chain-lightning, and much to my astonishment, for I did not expect such speedy action, found the six weeks old calf moribund. It seemed as if "fire had struck it" it had fallen away so. All the morning it had been severely constipated and strained ; its muffle was dry as parchment and covered with a whitish mass-like salt ; breathing had been very rapid ; had not eaten since inoculated so far as known ; would drink frequently, but little at a time.

NECROSCOPY ON CALF KILLED WITH TICK-GERM CULTURE.

As the animal was moribund, waited until quiet, then at once made autopsy. Skin carefully removed, "blood cultures" made, as directed, from the axillary vien. Took cultures from heart's blood, liver and spleen. Pieces of organs at once put into alcohol before the animal was cold. Sub-cutis somewhat emaciated; vessels very much engorged ; very many petechiæ scattered about, and an occisonial diffuse hemorrhage. Blood excessively thin, though dark colored, as in all previous cases, as is the great characteristic of the southern cattle plague, it resembled some watery dark-red dye stuff, far more than blood. This is best seen by letting it run over the fingers; it thickened, but formed no solid coagulum on exposure to the air while the autopsy was being made ; also reddened very slowly. Superficial lymph glands intensely swollen. Costal peritoneum and omentum dotted with innumerable petechiæ and ecchymoses; about a quart of straw-colored fluid in abominal cavity; serosa of small intestine of a diffuse dark-red, dotted by great numbers of still darker petechial and more extensive and less sharply defined hemorrhagic centers. Large intestine of a more grey color, with extensive diffuse hemorrhages and very few of a small circumscribed character. Mesenterial lymph-glands immensely swollen, œdematous, vessels engorged—many hemorrhagic centres in substance. Spleen excessively swollen and full of blood. Liver much swollen at base. Acini very much disturbed and mostly of a lustrous yellowish-grey color ; cut surface dry and anæmic ; gall capillaries beautifully injected and very prominent ; gall-bladder so distended as to resemble a blown-up bladder. Outside of second and third stomach fairly dotted with petechiæ, with some swelling of inside likening and a scattered hemorrhage here and there. Mucosa of fourth stomach excessively swollen, of a diffuse dark-pink-red color relieved by numerous petechiæ ; ecchymoses and more extensive and less limited hemorrhages of a much more intense color : a profuse greyish viscid coating covered the whole mucosa. Towards Pylorus, the coating was intense yellow, while the underlying tissues were deep red: mucosa of duodenum corresponded thereto; jejunum more and more red towards ileum, which was again intensely red ; follicles and patches markedly swollen. Lungs normal, except punctiform hemorrhages scattered though Pleura ; some aqueous effusions in air tubes, mucosa of which some swollen, many petechiæ. Bronchical lymph glands same as mesenteric.

The "blood cultures" from the axillary vein were at once placed in thermostat to test and demonstrate their value to find what was present, and were found not only pure by plate controls, but also so replete as to probably explain the mystery to the uninitiated in the technique of field–investigation, the "slides from Texas Fever diseased cattle swarming with bacteria." The cultures from the heart's blood and organs, on ordinary media were left at room temperature, subjected to every possible control, all being tested side by side on eggs and potatoes with the original and giving the same results.

SECOND CALF, STUNTED JERSEY, SIX MONTHS OLD. INOCULATED WITH TICK-GERM CULTURES.

This calf was very sick, became very much emaciated muffle dry, and covered with a white scale as in the other case. It finally recovered and was eventually killed by an intraveinous injection of the bacillus of the corn-stalk disease, the results of which are detailed in an article on that disease.

Clinical observations :

Temperature Oct. 9, 11.40 A.M. - - - - - - - 39.80 C.
" " 10, 7 A.M. - - - - - - - - 40.00 C.
Drank a little, but ate nothing.
" 11th, 7 A.M. - - - - - - - - 40.60 C.
Ate a few oats and drank little.
" 12th, 7 A.M. - - - - - - - - 41.20 C.
" 12th, 8 P.M. - - - - - - - - 42.60 C.

First extrement passed since the 9th, very dry and lumpy, with blood clots on it. Urine very dark red.

Temperature 13th, 7.30 P.M. - - - - - - - - 41.30 C.

Excrement very hard, passed in small quantities covered with slime and blood. Urine still dark-red. Calf not eating anything to speak of, respiration quick and calf getting emaciated, cannot get up without help.

| | | | | |
|---|---|---|---|---|
| Temperature | 14th, | 7.35 A.M. | - - - - - - - . | 40.10 C. |
| " | " | 8 P.M. | - - - - - - - - - - | 40.40 C. |
| " | 15th, | 6.10 A.M. | - - - - - - - - - | 40.35 C. |
| " | 16th, | 7 A.M. | - - - - - - - - - - | 39.80 C. |
| " | " | 8 P.M. | - - - - - - - - - - | 40.10 C. |

From this time on the animal slowly recovered. While sickest, some of the hard manure covered with slime was taken to the laboratory, and a small amount, not larger than a good sized pin's head, was rubbed up with sterlized water and ¼ ccm. was injected under the skin of a full-grown guinea-pig. It died in less than 24 hours, being dead, though not quite cold, when we got to the labortory the next morning, the exact time cannot be given. Pure cultures of the original germ injected into the calf were obtained from the blood and plate cultures from the manure gave a preponderance of the same germ.

About this time I went to Chicago and was away until about Dec. 10.

On Dec. 31, '91, I tested the original cultures from the tick in a guinea pig, and very much to my surprise, some unknown factor had materially mitigated their virulence.

This guinea pig died at 6 P.M. on the 9th of January, '92 ; pure cultures of the same germ were again obtained from its blood.

January 2, 1892. Two more guinea pigs were again inoculated from the same culture from the ticks as that used December 31, 1891, thinking there might possibly have been some unknown power of resistance in the first one. The first of the mdied January 6, 1892, 10 A. M., and the other the next day. Pure cultures of the true germ obtained from both.

Thinking now it must be in the media I tested all the bouillon in the laboratory made during my absence, and found it so acid, though not decidedly so, but not neutral by any means, that it was all thrown away, and new bouillon made under my personal supervision and the cultures from the guinea pigs at once transferred to it.

That the decrease in virulence must have been due to the influence of the unsuitable bouillon is shown by the next tests of the cultures which were made.

April 5, 1892. Three field mice received one drop subcutaneously in the thigh. All three died on the morning of the 6th. Pure cultures obtained from the heart's blood of each.

April 30, 1892, 10 A. M. Inoculated two old guinea pigs with ⅛ ccm. of the 33d germation from the tick, including the anima transfers; bouillon transfers to new material every seventh day. Both died on the morning of May 1st, and pure cultures were obtained from the heart's blood of each.

Certainly we have here sufficient evidence to conclusively demonstrate

1. That one species and one only of bacteria can be isolated from the blood and organs of cattle diseased with the southern cattle plague or Texas fever.

2. That the same micro organism has been clearly demonstrated to have been present in the blood and fæcal discharges of ticks taken from the bodies of southern cattle.

3. That the same organism derived from ticks can kill cattle when injected into the blood, as well as cause a disease in them presenting both lesions and symptoms of Texas fever.

4. That the manure from such cattle was repletely infested with same organism.

5. That the same micro-organism is in the tissues of all the animals that died or were killed on account of this disease as a natural infection, as well as such as were killed by experimental inoculation.

I now ask every unprejudiced reader or competent investigator, is there a single link wanting or a fracture in the chain of evidence in any direction that the southern cattle plague is not only caused by bacteria, but by the one here described? Is not the evidence in full accordance with the Koch postulates even when adhered to in the most orthodox manner?

Do not these investigations completely demonstrate that the methods of the government investigators must have been in the mildest sense of the term unscientific and hence totally unreliable; and that their dictum "Texas fever is a disease not caused by bacteria," is absolutely erroneous? That their final conclusion, "no ticks no Texas fever" must be equally so, though the ticks may or can, like the southern cattle, through their fæcal discharges, infest our northern pastures and thus our cattle are infected?

The dictum must still read: Ticks or no ticks, the southern cattle plague is caused by bacteria. Protozoa do not cause the disease, but if present, which is much to be doubted, are an accidental invasion.

This is another "humbug" created by the investigators of the agricultural department blown to the winds. It is to be wondered if any of them will again dare to face the verdict of the scientific world with the assertion that:

"I am prepared now to say that Texas fever is not a bacterial disease. There are no peculiar bacteria to be found in the blood, spleen, liver or other organs. All the illustrations which have been published showing preparations of blood from Texas fever animals swarming with bacteria, and sections of tissues showing the same micro-organisms, are misleading and may be put aside as of no value to the student. The blood and tissues do not present such appearances when properly examined." *Sic!*

Dr. Paul Paquin, late State Veterinarian of Missouri, and Investigator of Animal Diseases at the Agricultural Experiment Station made some investigations of Texas fever and published them in a bulletin dated May, 1890. I most cordially wish that I could speak more favorably than I can of this work of Dr. Paquin's, for I know he worked diligently and honestly. That he differs from me somewhat does not bear on the case at all, because differences in opinion will sometimes occur to everybody. It is only those who maliciously attack my work, or interfere with my desire to serve the public, or those who ignorantly support them, for whom I have no mercy and who excite my ire to a degree that will not permit of the usual language used in such discussions. I will simply give a few brief quotations from Dr. Paquin's Bulletin. The reader must take them at their own value. "Thirty-three different specimens from twenty different southern cattle of several counties in Texas and the Indian Territory were analyzed. In all of them without exception were found, after a long and tedious search, germs that could be plainly seen and easily stained. . . . In the blood and bile germs were frequently found in a motile condition. They would roll on themselves, bend to and fro, move from place to place, etc.

"The germs of Texas fever are found in all southern cattle coming from infectious grounds, not only in their manure, but in their blood, liver and spleen.

"That the germs in fresh excretions of southern cattle remain harmless twenty to fifty days, and in a cool shady place much longer."

I sent Dr. Paquin the few remaining slides of my cultures and tissues while I was in Chicago, of which he writes in his bulletin. He thinks that which I call the mature form, of which I am certain I'm correct, is not. Here is what he says:

"Dr. Frank S. Billings has grasped and explained better than any the significance of at least one form of the germ and understood much that investigators before him misunderstood, and has foreseen largely the possibilities and properties of the parasite. Judging from researches necessarily limited by time and circumstances, Dr. Billings concluded that in their mature form the germs presented a diameter of about twice that of their transverse, that they are very small, ovoid and have rounded extremities."

"Pure cultures can be obtained from the liver, spleen, kidneys, etc., of southern cattle of the infectious districts.

"The ticks full of blood from infected southern cattle may, it seems, scatter the germs on our lands, though ticks of themselves cannot convey the disease.

"With Billings I have found the germs motile, but only in certain forms. In drop cultures, as well as infected fluid or pulp from diseased subjects purposely killed, I have found this to be the case.

The trouble with Paquin's work is, that neither of his illustrations have a picture of a pure culture of anything, though he notes the ovoid germ, called by me the mature form (the ovoid-belted pole stained bacillus), nor have any accurate descriptions of any one organism been given. In fact justice compels me to say, that he does not so describe anything as to warrant the conclusion that he ever had a pure culture of any one organism in his possession. We have not the briefest description of developments in and on different media by which to make any comparisons so that his work is valueless regarding any specific germ, and only valuable in showing that bacteria are present in the blood of southern cattle, and by inference that he had under observation the same germ of which I sent him slides of cultures and tissues.

One thing surprises me, and that is that he should have had any difficulty in cultivating these organisms, for none grow easier and at room temperature on all the ordinary media when exactly neutral, or slightly, though not strongly alcaline. That they are a little difficult to catch in solid cultures from the heart's blood is true, and that is what brought me first to the "blood cultures" made at once as I have detailed, and taught me their great value, but if a large number of freshly prepared agar tubes are inoculated, some will develop. In neutral bouillon, however, I have never known the heart's blood to fail when several good-sized drops have been directly introduced. In fact, I never think of going into the field unless supplied with my "blood-culture," bottles and bouillon flasks. The solid media are less reliable and can be used late in the laboratory, though I always take some agar tubes with me also, but do not always use them when it is inconvenient, prefering to taking a piece of organ with me, wrapped as directed, and inoculate the tubes under cover. On this question of cultivation, Paquin surprisingly says: "No part of our work has offered more and greater difficulties than the artificial cultivation of the germs of Texas Fever. Beef-broth peptonized, solidified with agar or gelatine, or in a liquid state; blood serum, egg-albumen, potatoes, artificial lymph and several other media have been tried. We have found that they develop best in a mixture of artificial lymph "with beef broth." p. 11. I am sorry to say that I must flatly contradict every word of the above, as it is as emphatically contradicted by all my experiences, except regarding the artificial lymph, which is an article I know nothing about. In that regard I would most earnestly warn any young investigators against "running after strange gods." If they ever read the Bible they will know where that will lead to. Stick to the natural media and the proven media, but above all, study what natural media are and endeavor to imitate them all possible, for our artificial imitations will be found unnatural enough. This curiosity hunt, to see what germs will do under the most unnatural conditions, has led to any amount of unnecessary confusion, and should be religiously avoided in my opinion. As I have so often reiterated, the only inviolable point of diagnostic value is that a given germ is invariably found in all cases of a well-known and long recognized specific disease, and can be cultivated therefrom in any one medium, and the disease reproduced in the same species by inoculation. That is reliable testimony. Even though we cannot as yet cultivate the organism, but can diagnosticate it in the blood or a tissue, and with that blood or tissue again reproduce the disease in the same species, and again find the germ as before—that testimony is equally reliable.

### THE PERIODS OF EXPOSURE AND INCUBATION IN SOUTHERN CATTLE.

In the investigation of any infectious disease, but more especially one presenting so many striking phenomena as does the clinical history of this disease in our northern climate, one must be very cautious and differentiate very sharply between what may be called the period of exposure and that of incubation. The former, exposure, must naturally be very uncertain and more or less influenced by external conditions; while the latter, incubation, must present quite constant characteristics, being only influenced by the receptivity of the animal organism to infection, the physiological adaptation of the tissues of its organism to the biological necessities of the inficiens proper, the germs, and the degree of virulent activity, toxic or disease producing qualities, which the bacilli produces.

The period of incubation is the time which elapses from the first entrance of the specific inficiens into a susceptible organism to the time when the first physiological disturbances can be discovered, which in general would be a rise in temperature. It begins at once with the inoculation of the animals. In cases of natural infection we cannot control the clinical course of any disease with any such exactness, for the germs gain access to the animal body generally in small quanities and often at intervals, so that we know nothing of this presence until manifested by positive phenomena. In Texas Fever, for instance, the period of incubation under natural conditions is probably but a few days, perhaps 5 or 6, at least it is under fifteen days. Cattle have been seen to go into a pasture known to have been infested by southern cattle, and to have been first observed to be ill in 20 days from that time, as in a case at Tekomah, Neb., in 1887. The period of exposure, to illustrate by this disease, is the time elapsing from which the southern cattle were first introduced to that in which the disease first appeared in the natives, a most uncertain thing, varying as we have seen from thirty-three days in the shortest instance, to ninety in the longest, and averaging 59 80-100 days—dryness and temperature are the two primary factors exerting an influence on this period in the disease.

*An outbreak of Southern Cattle Plague in Northern Cattle, due to other Northern Cattle which were infected in a pasture infected by Southern Cattle.*

It has always been asserted that northern cattle infected with the southern cattle plague through the agency of Texans or other southern cattle could not possibly play any part in the further extension of the disease to other northern cattle. A true statement of the conditions would have been to have said that no case of the disease had ever been known to occur in which diseased northern cattle had taken any part. It has been in my good fortune to have not only seen, but to have studied such a case through its entire history from the original arrival of the southern cattle in the North to the primary outbreak in northern cattle due to infection on the pastures infested by the Texans, to a second outbreak caused in other northern cattle by the first natives infesting pastures after they were themselves diseased, on which no southern cattle had ever been and the second lot of diseased northern cattle had never been, nor any southern cattle. Notwithstanding that the Government investigators have fitted their "tick protozoon" hypothesis to this case also by the probably correct assertion that tick development goes on until cold weather stops it, still there were no ticks on the cattle on which I made autopsies in the second outbreak. So much I know! Whether or not there were any on the balance of the second infected native cattle I do not know.

The assertion that diseased natives caused and did infest their own pastures and cause a second outbreak in natives, met with the usual reception of any publication of the hitherto unknown or unobserved. It was in general flatly denied and contradicted as "impossible," the objectors forgetting that it is the "impossible" that is so often occurring and upsetting the most positive opinions of ignorance. This observation is, I think, the only one I have recorded which has not been contradicted by the Department of Agriculture; most probably because their "tick-protozoon" hypothesis was somewhat supported by it.

This second outbreak in natives occurred at Tekomah, Nebraska, in 1887. The cycle of events were as follows: Eleven hundred Texas cattle were imported into Nebraska on the 30th and 31st of March, 1887. The first indication of the disease in Northern cattle was observed in 21 town cows which were placed in the pasture where the Texans had been, from April 1st to April 15th, but were not at the time, after the grass had well grown, sometime about May 1st, 1887. These cows began to die about July 1st, and only one remained. Not a Texas steer was ever ill during the entire period For these cows the period of exposure and incubation, taken as one, was 90 days, as recorded.*

Now as to the second outbreak: On Sunday, June 19th, 1887. Twenty-four Northern steers, which had never been near a Texan, nor on grounds where one had been, escaped from their pasture, some six miles from town and were wandering about, and for safety, were thoughtlessly placed in a pasture where some 600 of the original Texans had been for about two weeks, that is from April 1st to 15th. In this pasture, these twenty-four natives remained, and grazed over night only. The next morning they were taken by their owners and again driven to their own pasture six miles distant, where there were 114 of their companions—138 in all being in this field.

They were first found sick on July 9th, and twenty-three of the original twenty-four became ill; two remained well or recovered.

* For fuller details the reader is referred to the earlier editions of this work.

Period of exposure and incubation about twenty days. Twenty-one died.

No further deaths, nor any cases of illness were seen in the balance of this herd (the 114 companions of the twenty which broke out of the pasture and were placed in that where the Texans had been) until September 21st, when the owners found several dead in the field. This was just fifty-two days from the time the twenty-four steers were seen to be sick after their return to this pasture after being placed in that on which the Texans had grazed, or been, from April 1st to April 15th. It will be seen how nicely this fact fits into the cycle of development of ticks, "from forty to sixty days," I am told.

Not a Texas steer had ever been inside the fence of this pasture in which this second outbreak in natives occurred.

The Texas cattle could not possibly have been the direct cause of this second outbreak in native cattle, though indirectly the cause of it having been the vehicle by which the bacilli were brought from their native soil in Texas to the first pasture where the first twenty-four steers became infested with them.

We want some proof of this and can find it easy and at hand.

In neighboring pastures, but seperated by two wire fences and a wide road, where two of the original lots of Texas cattle. They had been in this pasture since May 15th. In one field were nearly 600 of these Texans and one native which had been with them since May 15th, and all had been well since that time. In the next field were 200 Texans and about sixty native yearlings which had also been with them from May 15th to the 10th of August, when thirty of the yearlings were removed to another pasture. We shall refer to these thirty yearlings again soon. Now all these cattle, the 200 Texans and sixty yearlings, remained well up to August 10th, though two lots of native cattle which had been placed on pastures where the Texans had been from April 1st to 15th, had previously died. The 200 Texans and 30 yearlings left with them after August 10th, also remained well. Again, other lots of the same Texans had been pastured with natives ever since the middle of May without a single case of disease occuring among either natives or Texans.

These facts, then, go to show that from May 15th, 1887 to the end of the grazing season, that every possible opportunity had been given for native cattle to become infected through the agency of these Texas cattle and that the latter had been absolutely harmless during that time, though in the short time from April 1st to

15th, they had profusely infested pastures, when no natives were among them, that were pestiferously dangerous to natives after the middle of May the same year, though not a Texan had been on these pastures since April 15th.

These facts can be verified by any doubting person by suitable inquiries of Mr. Templeton and other owners of the Texans at Tekamah, Nebraska.

It seems to me that the above unquestionable facts in the history of these outbreaks of southern cattle plague at Tekamah, Nebraska, in 1887, completely upset the "Natural-tick-ova-hatched tick-protozoon" theory of the Aguricultural Department.

What were the ticks doing all this time, from May 15th to 1st, on some 1,100 Texans, among which, large numbers of native cattle were pastured? Certainly the season for inoculation and hatching was the favorable one. On particular inquiry of Mr. Templeton, he assures me, that there were still ticks on the Texans, and some on the natives during this time, though the latter all remained free from disease in every form.

Again, I questioned the cattle owners around Tekamah most sharply, and every one coincided in most positively asserting, that there was not a single case of infection in a native in or about Tekamah, that occured from any pasture, trail, or road-way on which the Texans were, or over which they were driven subsequent to April 15th, that is, fifteen days after their arrival in Tekamah.

Every place where they were prior to that date caused the infection of natives. This is especially true of the two fields where the Texans were herded from April 1st to 15th.

All these facts not only put a quietus on the tick-protozoon theory, but most positively demonstrate that the Texas cattle could have had no direct connection with the second outbreak of the southern cattle plague; that which was caused in the 114 natives on September 21st, by the 24 natives of the same herd which got out of their pasture on June 19th, and were placed where the Texans had been from April 1st to 15th.

The period of actual exposure and infection was in reality nearly the same in the second outbreak as the first, when we consider that the town cows were not put into the pastures where the Texans had been until about May 1st, and in this case the time which elapsed from the exposure on the pasture to the actual eruption of the disease must have been somewhat larger for climatic reasons. We shall again return to this topic.

This outbreak in the native 114 steers due to their twentyfour companions having been placed in a field where the Texans had been placed from April 1 to 15 is, fortunately, not the only evidence at hand of a second outbreak of this plague being caused by natives infected through the primary agency of Texans. Another occurred at the same time and place, at a distance from the first one.

A SECOND OUTBREAK IN NATIVES DUE TO NATIVES AT TEKAMAH, NEB.

During the July outbreak Mr. Templeton suffered more severe losses than any of his neighbors from the infection of his pastures by the Texas cattle, of which he was the largest owner. His loss was mainly in a herd of eighty cows. Thinking he could put a stop to the disease by removing the cows not yet sick from the field where they were, in the early days of the outbreak he took fifty of those remaining from the infected pasture and placed them on one distant and secure from all danger of infection from Texas cattle and watched them very closely. Nearly all died! The dead ones were drawn off out of this pasture and buried in a deep gulch at a distance where they were well covered. By the end of July this outbreak terminated and the pasture contained but a few recovered cattle and some horses, in which condition it remained until August 10, 1887.

Allusion has previously been made to a bunch of 200 Texas cattle that had been pastured with sixty native yearlings from May 15 to August 10, and that not only up to that date they all were well, but also that from August 11th to frost the Texans and thirty yearlings remained together and were free from disease. On August 10th Mr. Templeton took thirty of the sixty native yearlings and put them in the pasture where his isolated cows had died, and which had been free from disease for about two weeks, as stated above. He thought he was perfectly safe in doing this, as it was the general belief that the disease could be no further extended through infected northern cattle. The few animals on this pasture were not sufficient to consume the grass and so the thirty yearlings were taken from their companions and the 200 Texans and placed on this pasture where the cows had perished, and where no Texan had ever put hoof or dropped manure and hence "no ticks," and the protozooic accompaniment were possible, unless brought by the thirty yearlings themselves, but as their thirty companions still remaining with the Texans remained free from disease, we can dispense with "tick" in this emergency.

These thirty yearlings were "Polled-angus" heifers. They were removed to this pasture all well, on August 10, 1887, and on September 1st, or twenty days after, they were first observed to be ill and quite a number died. Here again we have a period of about fifty days from the time the original lot of native cows primary infected through the direct agency of the Texas cattle to the first cases of illness observed in second of native cattle.

These experiences show that under favorable climatic conditions about fifty days must elapse from the time southern cattle arrive in the North before native cattle can be expected to become infected, and that where a pasture has been already infected, and time enough elapsed for the distribution of the germs at a favorable time of year it takes about twenty days before native cattle exposed thereon begin to show serious signs of illness and deaths to occur.

(*To be Continued*).

